# Generation and Characterisation of Keratin 7 (K7) Knockout Mice

**DOI:** 10.1371/journal.pone.0064404

**Published:** 2013-05-31

**Authors:** Aileen Sandilands, Frances J. D. Smith, Declan P. Lunny, Linda E. Campbell, Kirsty M. Davidson, Stephanie F. MacCallum, Laura D. Corden, Lesley Christie, Stewart Fleming, E. Birgitte Lane, W. H. Irwin McLean

**Affiliations:** 1 Centre for Dermatology & Genetic Medicine, Division of Molecular Medicine, Colleges of Life Sciences and Medicine, Dentistry & Nursing, University of Dundee, Dundee, United Kingdom; 2 A*STAR Institute of Medical Biology, Singapore, Singapore; 3 Division of Cancer Research, Medical Research Institute, School of Medicine, University of Dundee, Dundee, United Kingdom; Sanford Burnham Medical Research Institute, United States of America

## Abstract

Keratin 7 (K7) is a Type II member of the keratin superfamily and despite its widespread expression in different types of simple and transitional epithelia, its functional role *in vivo* remains elusive, in part due to the lack of any appropriate mouse models or any human diseases that are associated with KRT7 gene mutations. Using conventional gene targeting in mouse embryonic stem cells, we report here the generation and characterisation of the first K7 knockout mouse. Loss of K7 led to increased proliferation of the bladder urothelium although this was not associated with hyperplasia. K18, a presumptive type I assembly partner for K7, showed reduced expression in the bladder whereas K20, a marker of the terminally differentiated superficial urothelial cells was transcriptionally up-regulated. No other epithelia were seen to be adversely affected by the loss of K7 and western blot and immunofluorescence microscopy analysis revealed that the expression of K8, K18, K19 and K20 were not altered in the absence of K7, with the exception of the kidney where there was reduced K18 expression.

## Introduction

Keratin 7 (K7) is a ∼55 kDa simple epithelial keratin which is primarily expressed in single-layered simple epithelia such as that found in glandular and ductal epithelia [1]. K7 is also expressed in certain stratified epithelia such as the bladder urothelium and within a discrete population of cells at the squamo-columnar junction in the stomach [2], [3]. Despite the widespread diagnostic application of K7 antibodies in the field of histopathology, very little information regarding the functional role of K7 *in vivo* exists - the lack of suitable mouse models combined with the fact that, to date, there have been no human diseases associated with mutations in the K7 gene, have all limited understanding of K7 function.

Unlike the epidermal keratins, whose functions are well defined due to their association with a large number of inherited skin disorders [4], the functions of the simple epithelial keratins ie. K7, K8, K18, K19, K20 and K23 have been more difficult to define [5]. Genetically engineered mice, either developed through gene targeting or overexpression of mutant keratin genes, have proved to be a useful tool in helping to understand the functions of the simple keratins and the careful characterisation of these different mouse models have helped in identifying human diseases not previously associated with keratin gene mutations [6]. For example, the phenotypic characterisation of various K8 and K18 knockout and transgenic mouse lines has been important in helping to demonstrate an association between predisposing KRT8 and KRT18 gene mutations in humans with various types of liver disease [7]. Pathogenic missense mutations in both of these genes have now been identified in patients with cryptogenic and non-cryptogenic cirrhosis, primary biliary cirrhosis and viral hepatitis [8].

The genes for the simple keratins K8, K18 and K19 have each been knocked out in mice and despite the fact that these keratins share overlapping patterns of expression, especially K8 and K18, the resulting phenotypes are quite different. The most severe phenotype is displayed by K8 knockout mice, which have a strain-dependent phenotype ranging from a highly penetrant mid-gestational lethality of K8 null embryos on the *C57Bl6* genetic background [9] to colorectal inflammation and hyperplasia on a surviving *FVB/N* genetic background [10]. In contrast, K18 knockout mice have a relatively mild age-related phenotype which is restricted to the liver and consists of the accumulation of K8-positive aggregates in hepatocytes [11]. Knockout of K19 does not lead to any obvious phenotype in mice [12], which is probably due to compensation by K18, but breeding of K19 knockout mice with either K8 or K18 null mice produces K8/K19 and K18/K19 double knockout embryos which die *in utero* [12], [13]. The failure of these double keratin-deficient embryos to survive has been attributed to fragility of trophoblast giant cells in the developing placenta caused by the lack of an intact keratin cytoskeleton [13]. Therefore in the placenta at least, simple keratins provide an essential structural role in maintaining the integrity of the trophoblast layer, much akin to the role played by the epidermally-expressed keratins which give structural support to the skin and its appendages.

In an attempt to understand better K7 function *in vivo*, as well as to increase the overall number of keratin knockout mice that are available for study, we used our previous experience with the mouse *Krt7* gene [2] to introduce a null mutation into mouse embryonic stem cells by gene targeting. By generating K7 deficient mice, the consequences of the absence of K7 on the development and differentiation of simple epithelia can be studied, the outcome of which might be useful in discovering hitherto unknown human disorders associated with *KRT7* gene mutations.

## Materials and Methods

### Construction of the Krt7 Gene Targeting Vector

**Figure 1:**
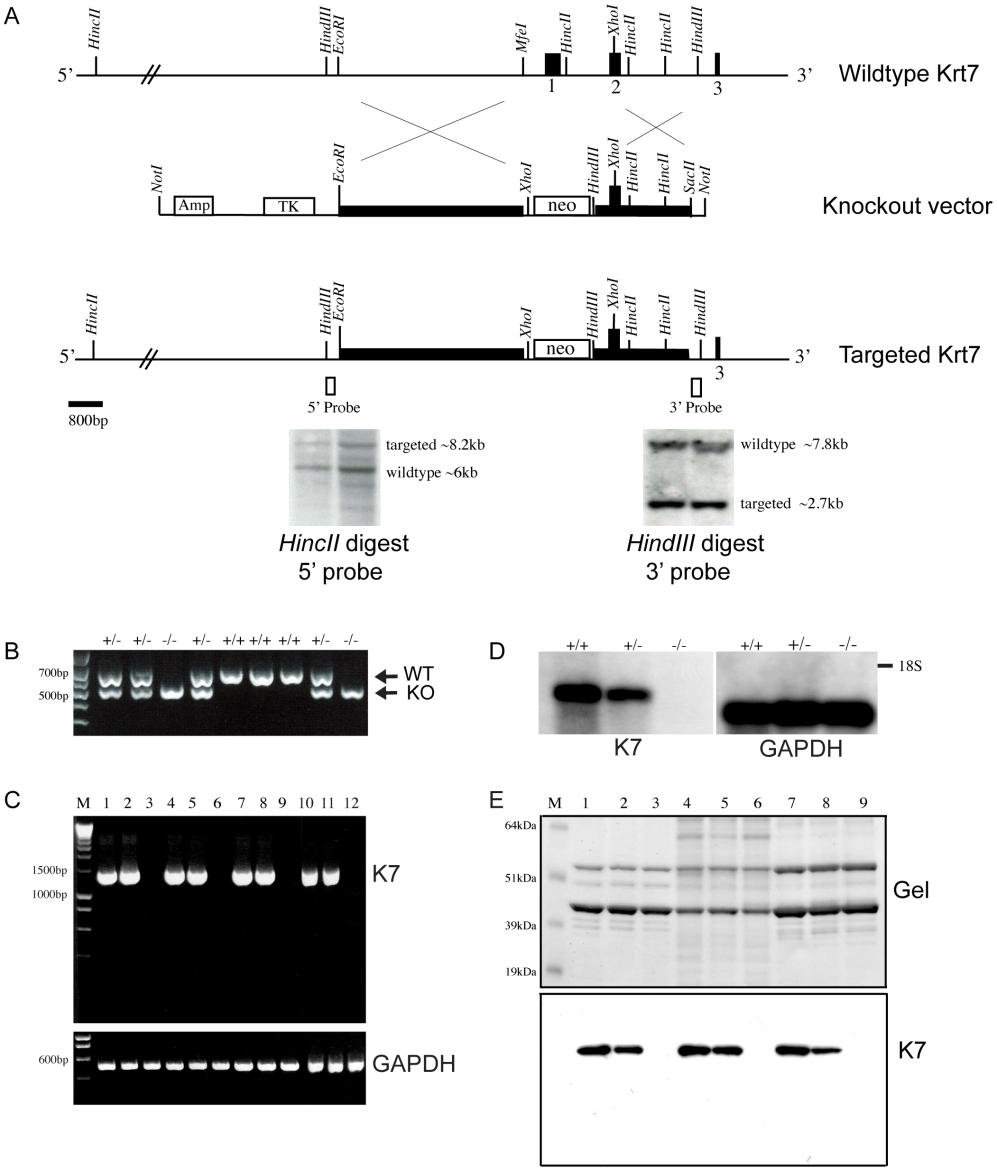
*Krt7* gene targeting strategy. A. Schematic diagram of the mouse *Krt7* gene and upstream sequences, only the proximal part of the gene encompassing exons 1 to 3 is shown. Filled black boxes denote exons 1, 2, 3. The long and short homology arms of the targeting vector are indicated by filled black rectangles. Restriction enzyme sites are as indicated and the open black boxes denote the locations of the DNA probes that were used for southern blotting at the 5′ and 3′ ends of recombination in targeted ES cells. B. Genotyping of K7 knockout mice, the wildtype allele is 743 bp, the targeted allele is 593 bp. C. RT-PCR analysis of cDNA from the bladder (lanes 1–3), lung (lanes 4–6), colon (lanes 7–9) and kidney (lanes 10–12) amplified with primers to full-length K7 (∼1.5 kb) and GAPDH (509 bp). Lanes 1, 4, 7 and 10 are from wildtype mice; lanes 2, 4, 6 and 8 are from heterozygote K7 knockout mice; lanes 3, 6, 9 and 12 are from homozygous K7 knockout mice. M = DNA size standards. D. Northern blot of bladder RNA from wildtype (+/+), heterozygous (+/−) and homozygous (–) mice detected with RNA probes to K7 and GAPDH. The position of the 18S ribosomal subunit is indicated by a black bar. E. Coomassie blue SDS-PAGE gel and western blot of cytoskeletal extracts prepared from the bladder (lanes 1, 2, 3), lung (lanes 4, 5, 6) and colon (lanes 7, 8, 9) of wildtype (lanes 1, 4, 7), heterozygous (lanes 2, 4, 6) and homozygous (lanes 3, 6, 9) K7 knockout mice probed with a C-terminal K7 antibody. M = molecular weight standards, sizes in kDa are as indicated.

The mouse *Krt7* gene was isolated from a PAC *129S6/SvEvTac* genomic DNA library, subcloned into pUC18 and completely sequenced [2]. To facilitate the construction of the K7 knockout vector, a 2063 bp PCR product which comprised the short arm of *Krt7* homology was amplified from the original pUC18 clone and cloned into pCR2.1 (Invitrogen). The amplification primers for the short arm of homology incorporated *HindIII* and *SacII* sites to facilitate the selection of targeted ES cell clones. *HindIII* and *SacII* double-digestion of the short arm of homology in pCR2.1 produced a ∼2 kb fragment which was subcloned into the *HindIII* and *SacII* sites of the targeting vector pNTKV-1906 (Clontech) to generate the construct pNTKV-1906/3′K7. pNTKV-1906/3′K7 was then digested with *EcoRI* and a 4148 bp *EcoRI/MfeI* restriction fragment (the long arm of homology) was subcloned into this site using blunt-ending cloning to generate the complete *Krt7* knockout vector (Figure 1). The targeting vector was linearised with *NotI* prior to electroporation into E14 mouse embryonic stem cells.

### Generation of K7 Knockout Mice

10^7^ E14 (*129P2)* embryonic stem cells were electroporated with 35 µg of linearised targeting vector and seeded onto mitomycin-C treated embryonic fibroblast feeder cells. Transfected ES cells underwent double-selection with the neomycin analogue G418 (Gibco), at a concentration of 200 µg/ml and with gancyclovir (2 mM). ES clones were screened by Southern blot analysis using DNA probes external to both the 5′ and 3′ ends of the *Krt7* homology arms. Microinjection of ES cell clones and generation of chimeric mice were performed as described previously [14]. Chimeric male mice were mated with *C57BL/6* female mice and germline transmission of the targeted *Krt7* allele was confirmed by PCR analysis of the resulting offspring. For continued maintenance of the line, K7 knockout mice were backcrossed onto the *C57Bl6* background. Genotyping of K7 knockout mice was performed using the following primers: forward primer 5′ CTA CTG GCC TCA GGA ATC TAG G 3′; reverse primer 1 5′ AAG AAC CGT GCG ACT GAG 3′ and reverse primer 2 5′ GAA TAT CAT GGT GGA AAA TGG C 3′ to generate PCR products of 743 bp (wildtype allele) and 593 bp (knockout allele). PCR conditions were as follows: 1 cycle at 94°C for 3 minutes followed by 40 cycles of 94°C (30 sec); 58°C (30 sec); 72°C (1 minute) and a final extension cycle of 72°C for 5 minutes.

### Southern Blotting

DNA from either targeted ES cells or mouse tail tips was digested overnight with appropriate restriction enzymes and then separated on 1% (w/v) agarose gels. DNA gels were transferred to Hybond N+ membrane (GE Healthcare) overnight. DNA probes for Southern blotting were non-radioactively labelled using fluorescein (GE Healthcare). Probes were hybridised overnight at 60°C then washed in 1×SSC/0.1% (w/v) SDS followed by 0.5×SSC/0.1% (w/v) SDS. The bound probes were visualised using an anti-fluorescein antibody conjugated to alkaline phosphatase followed by chemilluminescent detection.

### Northern Blotting

mRNA was purifed from mouse tissues using the QuickPrep Micro mRNA purification kit (GE Healthcare) according to the manufacturer’s instructions. mRNA samples were separated on 1.2% (w/v) formaldehyde-agarose gels with MOPS running buffer and transferred overnight onto Hybond N+ membrane. Blots were incubated overnight at 68°C with an *in vitro* transcribed dioxygenin-labelled K7 RNA probe corresponding to exons 6–9 of the murine K7 cDNA. Following probe hybridisation, blots were washed twice in 2×SSC/0.1% (w/v SDS) at room temperature (5 minutes per wash) followed by 2 washes in 0.2×SSC/0.1% (w/v SDS) at 68°C (15 minutes per wash). The bound probe was detected using a sheep anti-digoxygenin antibody (Fab fragments) conjugated to alkaline phosphatase (Roche) followed by chemilluminescent detection.

### Quantitative RT-PCR

Tissues were mechanically disrupted using the Qiagen TissueLyser LT. Total RNA was extracted using the Qiagen RNeasy extraction kit according to the kit protocol. In-column treament of the RNA with DNase was performed to remove genomic DNA contamination. 2 ug of RNA was reverse transcribed into cDNA using the High Capacity cDNA Reverse Transcription kit (Applied Biosystems). 0.5****µl of cDNA was amplified in a 20 ul reaction using pre-designed Taqman® Gene Expression assays for *Krt7* (Mm00466676_m1), *Krt8* (Mm04209403_g1), *Krt18* (Mm01601704_g1), *Krt19* (Mm00492980_m1) and *Krt20* (Mm00508106_m1) and ran on a 7900HT Fast Real-Time PCR system (Applied Biosystems) following the manufacturer’s recommended protocol. A Taqman® probe for mouse GAPDH was used as the endogenous control. Relative quantification (RQ) using the Comparative C_T_ method was determined using the RQ Manager 1.2.1 software (Applied Biosystems).

### Immunofluorescence Microscopy

Mouse tissues (a minimum of 3 mice per genotype) were embedded in OCT (Agar Scientific) and immediately frozen in liquid nitrogen. 10 µm sections were fixed in methanol:acetone (−20°C) and blocked with 10% (v/v) normal goat serum (Sigma) in PBS buffer containing 0.1% (w/v) BSA. Primary and secondary antibodies were diluted in PBS buffer containing 0.1% (w/v) BSA and incubated for 1 hour at room temperature. Sections were extensively washed between antibody incubations with PBS. Primary antibodies were detected using appropriate Alexafluor-488 or Alexafluor-594 conjugated-secondary antibodies (Molecular Probes). Following antibody labelling, sections were mounted under glass coverslips with Hydromount containing 2.5% (w/v) DABCO (Sigma).

### Immunohistochemistry

Mouse tissues (a minimum of 3 mice per genotype) were immediately fixed in 10%(v/v) Gurr neutral buffered formalin (pH 7.4) for 48 hours before dehydration and embedding in paraffin wax. 5 µm sections were stained with Mayer’s haematoxylin and eosin. Tissue sections were examined by a clinical pathologist experienced in the histological analysis of mouse tissues. For antibody staining, high-temperature antigen retrieval was performed overnight by incubating sections with 0.01 M citrate buffer. Endogenous peroxidase was quenched by 1% v/v H_2_O_2_ in PBS for 30 minutes. Sections were blocked with either goat or rabbit serum (10% v/v in PBS) depending on the host species of the secondary antibody. Primary antibodies were incubated for 1 hour at room temperature and were detected by HRP polymer anti-mouse or anti-rabbit Envision antibodies (DAKO). Sections were developed using DAB substrate (DAKO) then counterstained with Mayer’s haematoxylin.

### Gel Electrophoresis and Western Blotting

Tissues were either processed immediately or snap-frozen in liquid nitrogen for storage at −80°C. Cytoskeletal-enriched extracts were prepared from tissues by homogenisation in high-salt extraction buffer (20 mM Tris-HCl pH7.4, 0.6 M KCl, 1% v/v Triton X-100 and protease inhibitor cocktail (VWR International)) followed by extraction in 9 M urea/1% v/v β-mercaptoethanol/10 mM Tris-HCl (pH8). Protein samples were run on 4–12% w/v gradient Bis-Tris Novex gels (Invitrogen). For western blotting, gels were transferred to nitrocellulose paper and blocked in 5% (w/v) non-fat milk powder in TBS buffer containing 0.2% (v/v) Tween-20 (VWR International). Primary and secondary antibodies were diluted in 1% (w/v) BSA in TBS buffer containing 0.06% (v/v) Tween-20. Primary antibodies were detected using goat anti-rabbit, goat anti-mouse or rabbit anti-rat immunoglobulins conjugated to horseradish peroxidase (DAKO). The antigen-antibody complex was then visualised chemilluminescently using luminol (Fluka) as substrate.

### Antibodies

For the detection of K7, an affinity-purified rabbit polyclonal antibody raised against a C-terminal peptide of mouse K7 was used [2]. Mouse K8 was detected using rat monoclonal antibody Troma I (Developmental Studies Hybridoma Bank, Univ. Iowa). Mouse K18 was detected using an anti-K18 monoclonal antibody (clone Ks18.04; Progen, Germany). Mouse K19 was detected by immunofluorescence staining using rat monoclonal antibody Troma III (Developmental Studies Hybridoma Bank, Univ. Iowa) and by mouse monoclonal antibody LP2K for western blotting. Mouse K20 was detected either with an anti-cytokeratin-20 monoclonal antibody (SPM140; abcam®) or for immunoblotting, mouse monoclonal antibody XQ1 [15]. For western blotting of cytoskeletal extracts, a mouse monoclonal antibody to β-actin (clone AC-15; Sigma) was used as a control to monitor protein loading. Ki-67 was detected using a mouse monoclonal antibody (clone MM1; Leica Biosystems). Uroplakin 3a was detected using a rabbit polyclonal antibody (H-180; Santa Cruz Biotechnology).

### Statistical Analysis

Two-tailed Students t-test was used was for pairwise comparisons of data sets and a *p* value of <0.05 was considered to be statistically significant.

### Ethical Considerations

All work involving animals was approved by the University of Dundee ethical review committee and all scientific procedures were performed under project licence authority (to WHIM and EBL) from the Home Office in accordance with the Animals (Scientific Procedures) Act 1986.

## Results

To generate K7 knockout mice, we replaced exon 1 of the *Krt7* gene and ∼270 bp of the proximal promoter with a neomycin resistance cassette (Figure 1A) in order to prevent transcripts originating from this locus. Homozygous K7 knockout mice, on either the original *129P2/C57Bl6* mixed genetic background or those on the inbred *C57Bl6* background, were born from heterozygous intercrosses at the expected Mendelian frequency of 1∶2:1 indicating that the absence of K7 did not affect embryonic development. Homozygous K7 knockout mice were phenotypically indistinguishable from heterozygous and wildtype littermates and both male and female homozygotes were fertile and reproduced normally.

**Figure 2:**
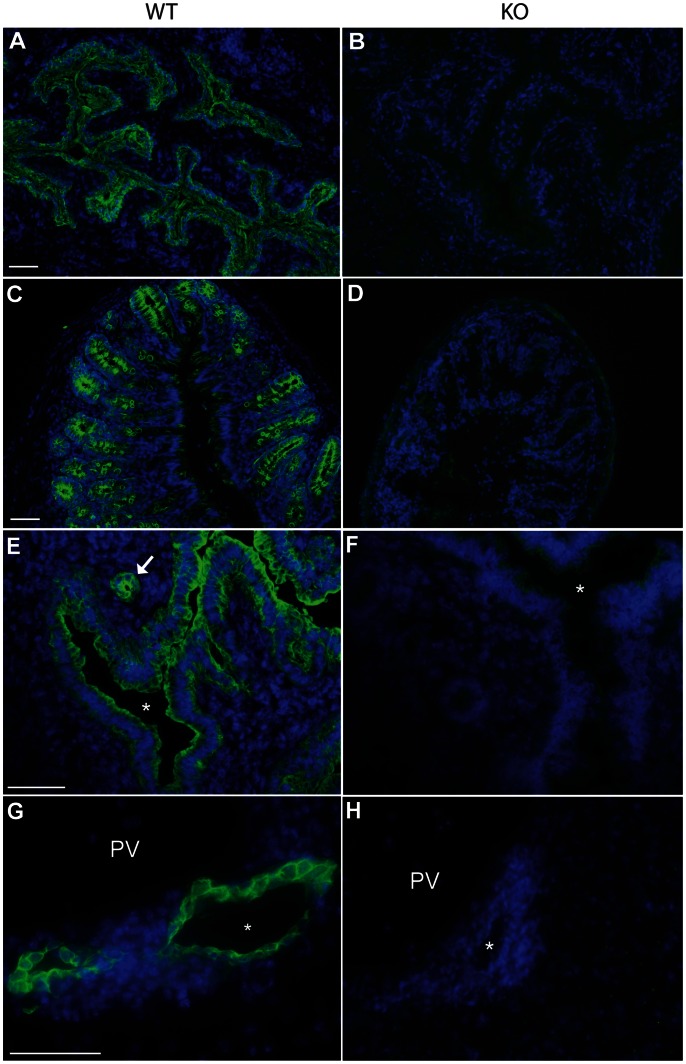
Loss of K7 expression in K7 knockout mouse tissues. Immunofluorescence microscopy of frozen sections of bladder (A,B), colon (C,D), uterus (E, F) and liver (G, H) of wildtype (A, C, E, G) and homozygous K7 knockout mice (B, D, F, H) stained with polyclonal antibodies to K7 (green). Nuclei are counterstained with DAPI (blue). The asterisks in panels E and F denote the lumen of the uterus. PV denotes the portal vein of the liver; the asterisks in panels G and H indicate the lumen of the bile duct. Scale bars = 50 µm.

In the mouse, K7 is primarily expressed in various ductal and glandular epithelia and is highly expressed in transitional epithelia such as the bladder urothelium [2]. Semi-quantitative RT-PCR analysis of cDNA prepared from the bladder, lung, colon and kidney confirmed the absence of *Krt7* transcripts in homozygous K7 knockout tissues (Figure 1C). Northern blotting of mRNA prepared from the bladder using the mouse K7 cDNA (exons 1–6) as a probe showed the absence of any K7 mRNA transcripts in homozygous mice whereas in heterozygous mice there was approximately half the amount of *Krt7* mRNA as compared to wildtype mice (Figure 1D). Western blotting of cytoskeletal-enriched extracts prepared from the bladder, lung and colon of homozygous K7 knockout mice showed no K7 protein (Figure 1E). In heterozygous mice, there was an appreciable reduction in the amount of K7 protein as compared to cytoskeletal extracts prepared from wildtype tissues. Immunofluorescence microscopy of tissue cryosections using a polyclonal antibody raised against the C-terminus of murine K7 confirmed the absence of K7 protein in homozygous K7 knockout mice (Figure 2 and results not shown). In heterozygotes, K7 staining was comparable to that observed in wildtype tissues (results not shown). Overall, these series of experiments demonstrated that our inactivation of the *Krt7* gene using gene targeting had been successful.

**Figure 3:**
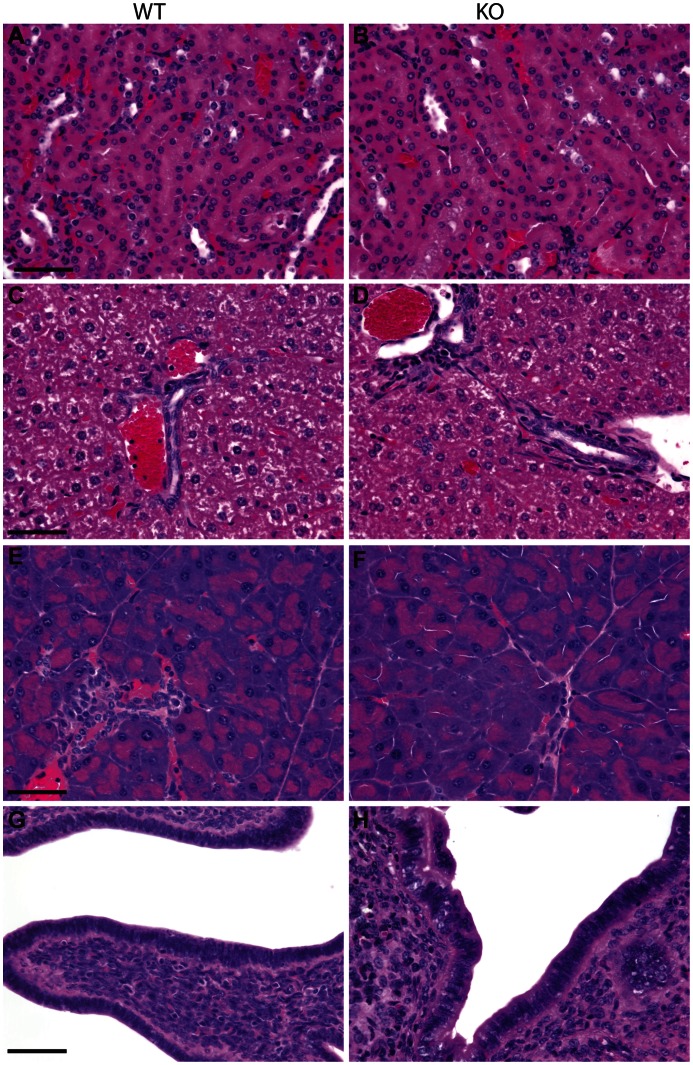
Histological analysis of K7 knockout tissues. Haematoxylin and eosin stained formalin-fixed tissue sections from wildtype (A, C, E, G) and homozygous K7 knockout mice (B, D, F, H). Images show the cortical collecting tubules of kidney (A, B), bile ducts in liver (C, D), pancreatic ducts (E, F) and columnar epithelium of uterus (G, H). Scale bars = 50 µm.

Based on our earlier study of K7 expression in the mouse [2], we undertook a comprehensive histological analysis of tissues from 6–8 week old homozygous K7 knockout mice (Table S1). We could detect no gross histological differences between the tissues and organs of homozygous K7 knockout mice as compared to wildtype littermates (Figure 3 and results not shown). Furthermore, the genetic background did not appear to influence these results since we could detect no histological differences between the tissues of homozygous K7 knockout mice that were from the original *129P2/C57Bl6* mixed genetic background and those that were maintained on a *C57Bl6* background (results not shown). We also considered the possibility that a phenotype could be late-onset, as has been shown for K18 knockout mice [11], therefore we performed the same histological analysis in a small number (n = 3) of homozygous K7 knockout mice from the original *129P2/C57Bl6* mixed genetic background that were over 1 year old in age (average age = 581 days; SE = +/−9 days), but we still could not detect any histological differences in the tissues or organs of these older mice when compared to their wildtype littermates (results not shown).

**Figure 4:**
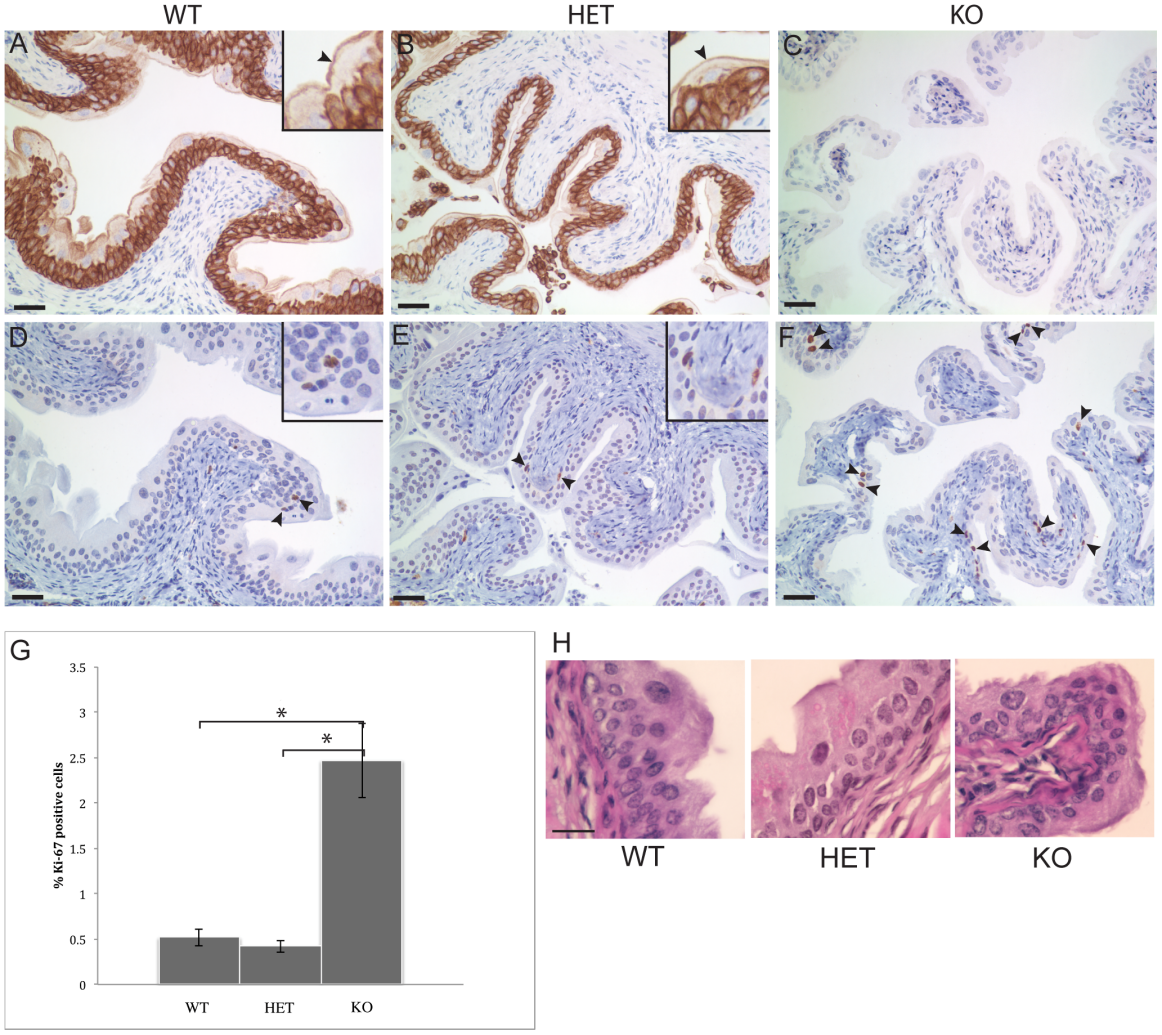
Loss of K7 is associated with hyperproliferation but not hyperplasia of the bladder urothelium. Immunohistochemistry of bladder sections from wildtype (A, D), heterozygous (B, E) and homozygous K7 knockout mice (C, F) stained with a rabbit polyclonal antibody to K7 (A, B, C) and mouse monoclonal antibody MM1 to the cell proliferation marker Ki-67 (D, E, F). Arrowheads and insets in panels D and E indicate Ki-67 positive nuclei in wildtype (D) and heterozygous K7 knockout (E) bladder. More Ki-67 positive cell nuclei can be seen in the bladder of homozygous K7 knockout mice (arrowheads in F). Scale bars = 50 µm. G. Graph showing the percentage of Ki-67 positive urothelial cells in wildtype, heterozygous and homozygous K7 knockout mice (5 bladders per genotype). For each bladder, 10 random images were collected and an average of 1480 (SD +/−300) urothelial cell nuclei were counted. Standard errors (SE) are indicated by the capped lines. * indicates a *p* value of less than 0.05 (WT *p* = 0.01; HET *p* = 0.007). H. H&E stained sections of the bladder urothelium of wildtype, heterozygous and homozygous K7 knockout mice. Scale bar = 25 µm.

Immunohistochemical staining of tissue sections with antibodies to the cell proliferation marker Ki-67 revealed normal epithelial cell proliferation in homozygous K7 knockout mouse tissues (results not shown) with the exception of the urinary bladder where there appeared to be more Ki-67 stained nuclei (Figure 4F). Counting of Ki-67 positive cell nuclei in the transitional epithelium of the bladder, the urothelium, revealed a proliferation index of around 0.5% in wildtype and heterozygous K7 knockout mouse bladder (Figure 4G) which is in close agreement with an earlier study by Farsund who reported a proliferation index in the normal mouse urothelium of around 0.4% [16]. In contrast, in homozygous K7 knockout mice, around 2.5% of urothelial cells were Ki-67 positive representing an approximate 5-fold increase in proliferation (Figure 4G) but despite increased proliferation, the bladder urothelium of homozygous K7 knockout mice showed no overt histological evidence of hyperplasia (Figure 4H). Immunohistochemical staining of bladder sections from K7 knockout mice with antibodies to uroplakin 3a, a differentiation marker of urothelial cells, was similar to wildtype mice indicating normal terminal differentiation of the urothelium (Figure S1).

**Figure 5:**
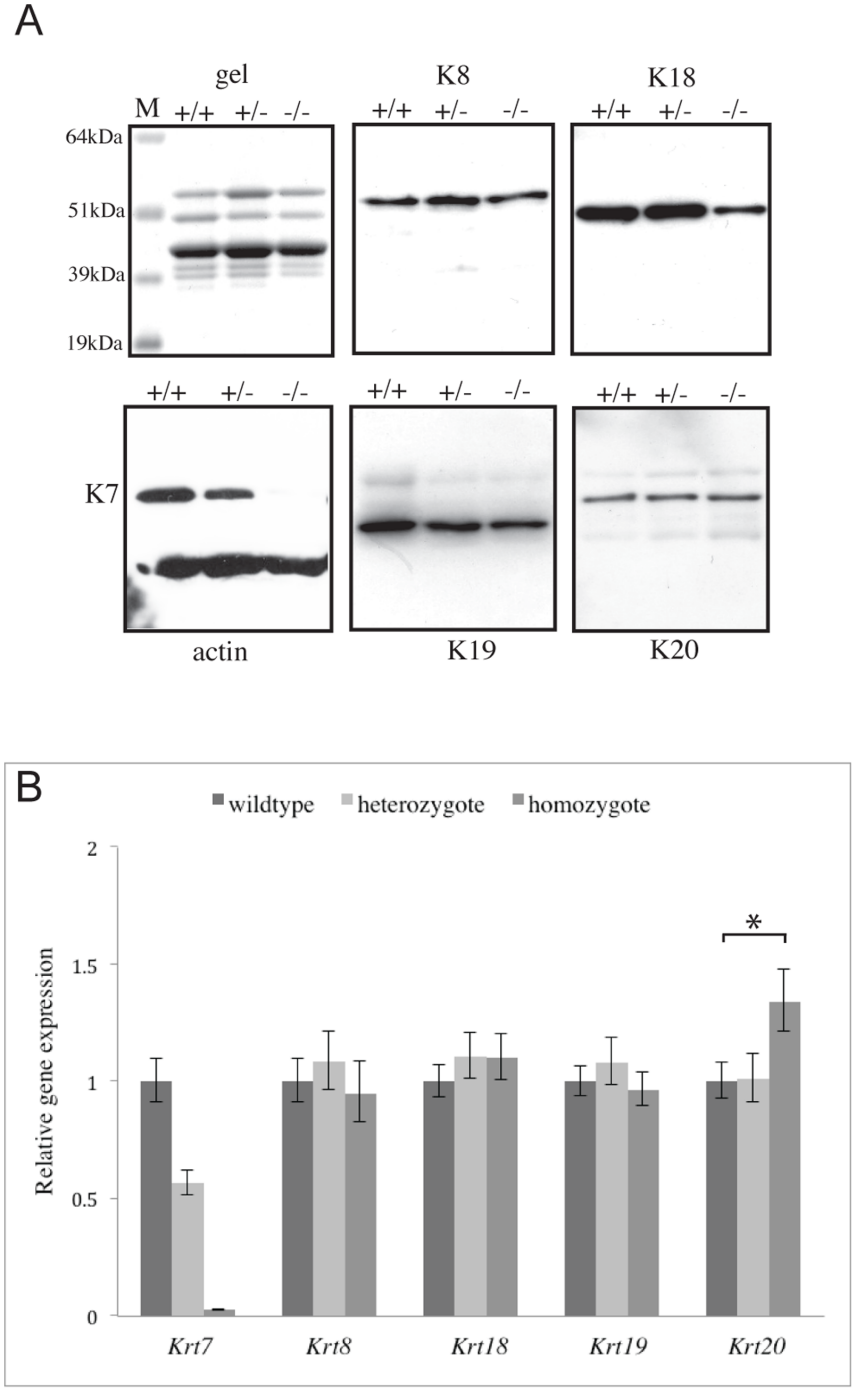
Simple keratin expression in the bladder of K7 knockout mice. A. Coomassie blue stained SDS-PAGE gel and western blots of cytoskeletal-enriched extracts prepared from the bladder, lung and colon of wildtype (+/+), heterozygous (+/−) and homozygous (–) K7 knockout mice probed with antibodies to K8, K18, K19 and K20. A monoclonal antibody to β-actin was used to monitor protein loading and was co-incubated with the polyclonal K7 antibody on the same blot. M denotes molecular weight standards, sizes in kDa are as indicated. B. Quantitative RT-PCR of bladder cDNA from wildtype, heterozygous and homozygous K7 knockout mice (five bladders per genotype) analysed with Taqman® gene expression probes to *Krt7*, *Krt8*, *Krt18*, *Krt19* and *Krt20*. GAPDH was used as the endogenous control to allow for normalisation between individual samples. Three experimental replicates were performed for each cDNA sample and the results for each of the five biological replicates per genotype were combined to determine the level of gene expression. The expression level of each gene in heterozygous and homozygous samples is shown relative to the expression of the wildtype sample. The error bars indicate the maximum (RQmax) and minimum (RQmin) expression levels for each gene as determined by the RQ Manager 1.2.1 software (using 99% confidence limits). * indicates a *p* value of 0.04.

Since the bladder urothelium was the only epithelial type that was affected by the absence of K7, and because K7 naturally exists as a heteropolymer *in vivo*, we then investigated the expression of the remaining simple keratins in the bladder. K18, K19 and K20 are all potential *in vivo* assembly partners for K7 in the bladder, meaning that their expression could be affected by the loss of K7, whereas K8 represents the remaining simple type II keratin in this tissue. Western blotting (Figure 5A) and immunofluorescence microscopy (Figure S2) showed reduced K18 expression in the bladder of homozygous K7 knockout mice although gene expression analysis showed this was not due to reduced *Krt18* mRNA (Figure 5B). K8 and K19 were not affected by the loss of K7 (Figure 5) but there was a significant increase in K20 mRNA expression in homozygous K7 knockout mice (Figure 5B). However a concomitant increase in K20 protein was not detected by western blotting (Figure 5A) or by immunofluorescence microscopy (Figure S2).

To continue our characterisation of K7 knockout mice, we extended our analysis of simple epithelial keratin expression to other tissues using double-label immunofluorescence microscopy using the K7 polyclonal antibody in combination with monoclonal antibodies to K8, K18, K19 and K20 and the results are summarised in Table 1. We confirmed some of the results using western blotting (Figure S3) although this method was not applicable to every tissue due to the restricted expression of K7 to certain cell types such as the ducts of the liver and pancreas. Based on these analyses we could not detect any changes in K8 expression in homozygous K7 knockout mouse tissues as compared to tissues from wildtype mice (Table 1 and Figure S3). Immunofluorescence staining suggested reduced K18 expression within the collecting tubules of the kidney of K7 knockout mice (Figure S4) whereas other tissues showed no difference in K18 expression (Table 1). Despite extensive co-expression with K7 in wildtype tissues, there was no difference in K19 expression in the tissues of homozygous K7 knockout mice as compared to wildtype tissues (Table 1 and Figure S5). K20 is even more restricted in its pattern of expression as compared to K8 or K19, and in wildtype tissues where it is expressed along with K7, it did not appear to co-localise with K7 except for in the bladder where there was co-localisation of K7 with K20 at the apical cell membranes of superficial urothelial cells (Figure S2). However, in the absence of K7, K20 still remained localised at the apical cell membranes of urothelial cells (Figure S2). In other K7 knockout tissues, K20 expression was unchanged (Table 1).

**Table 1:** Immunofluorescence analysis of simple keratin expression in K7 knockout mice.

Tissue	K7 expression	K8	K18	K19	K20
Bladder	Urothelium	=	reduced*	=	=
Liver	Bile ducts	=	=	=	ne.
Colon	Basal cells in crypts, goblet cells	=	=	=	=
Kidney	Collecting tubules & ducts	=	reduced	=	ne.
Lung	Alveolar & bronchiolar epithelium	=	=	=	ne.
Pancreas	Ductal epithelial cells	=	=	=	ne.
Duodenum	Brunner’s gland & specific cells in crypt	=	=	=	=
Stomach	Squamo-columnar cells	= ¶	= ¶	= ¶	= ¶

= intensity of staining and localization similar to wildtype tissue.

* confirmation by western blotting.

ne. no protein expression.

¶ glandular cell staining.

## Discussion

In this paper we have described the generation and characterisation of K7 knockout mice, one of the remaining so-called “simple” epithelial keratin genes to be knocked out in the mouse using conventional gene targeting. Although the absence of K7 protein was not associated with any pathological phenotype this is not an unexpected result given that K18 knockout mice, which also show secondary loss of K7, only developed a mild late-onset phenotype that was restricted to hepatocytes with no other associated pathology [11]. Compensation for the loss of K7 by other type II keratins, in particular K8 whose expression closely overlaps with K7 [1], provides the most likely reason for the lack of any overt phenotype in K7 knockout mice but overcoming this problem of functional redundancy, through the generation of a K7/K8 double knockout mouse for example, would be difficult since both genes are closely associated within the keratin gene cluster on mouse chromosome 15 and based on previous studies embryos lacking both K7 and K8 are likely to be non-viable anyway [12], [13].

Despite the absence of any pathology associated with the loss of K7, homozygous K7 knockout mice showed increased proliferation of the bladder urothelium.

Hyperproliferation is a feature of several keratin mouse knockouts and includes those which affect internal epithelial such as K8 [10] and K4 [17] as well as certain epidermally-expressed keratins such as K10 [18]. However, unlike these keratin knockout mice where hyperproliferation was associated with hyperplasia, there was no apparent urothelial hyperplasia in the bladder of K7 knockout mice. Unlike other types of epithelia such as the colon and epidermis, urothelial cells are characterised by their low proliferation index and longevity [16], [19]. Therefore the five-fold increase in urothelial cell proliferation that we observed in K7 knockout mice, although similar to the four-fold increase in keratinocyte proliferation observed in the hyperplastic epidermis of K10 knockout mice [18], may simply have been insufficient to produce a phenotype in this particular type of epithelium. It is not clear how the absence of K7 led to stimulation of the normally quiescent urothelium since histologically there was no obvious disruption to the urothelium such as loss of the superficial umbrella cell layer, nor any evidence of apoptotic urothelial cells. Moreover, there was no evidence of any inflammatory cellular infiltrate present within the urothelium or the underlying bladder mucosa and the expression of the differentiation-associated plaque protein uroplakin 3a, which is important for limiting transcellular permeability across the urothelium [20], was also normal in K7 knockout mice suggesting that the urothelial barrier was intact. Further study is therefore required in order to understand how the loss of K7 leads to changes in urothelial cell proliferation.

Our analysis of the fate of remaining simple keratins in K7 knockout mice suggests that K7 is required in part for the stabilisation of K18 *in vivo*. Unlike K18 knockout mice which showed complete loss of K7 [11], in K7 knockout mice K18 protein levels were only reduced but this appears to be a tissue-dependent effect since it was only noted in the bladder and in the kidney. In contrast to K18, the tail-less type I keratin K19, despite extensive co-expression with K7 in all of the tissues we examined, did not appear to be affected by the loss of K7 suggesting that K19 must be stabilised through pairing with another type II keratin, most likely K8. Although we only found minimal overlap between the expression pattern of K20 and K7 in the tissues that we examined, both proteins are strongly expressed in the bladder urothelium although they only co-localise at the apical surface of the terminally differentiated superficial cells where they contribute to a trajectorial keratin network that underlies the plasma membrane [21]. The upregulation of *Krt20* gene expression that we observed in the bladder of K7 knockout mice might be interpreted as an attempt to compensate for the loss of K7 protein from the sub-apical cytoskeleton in these superficial cells. Although we measured a significant increase in *Krt20* mRNA expression in the bladder of K7 knockout mice, we could not detect any concomitant increase in the amount of K20 protein. This disparity could simply be due to differences in the sensitivity of the gene expression assay versus western blotting which is only semi-quantitative. It is also possible that since we blotted cytoskeletal extracts which contain filamentous ie. assembled keratin, rather than total protein extracts which would have included any soluble keratin that was not incorporated into filaments, any additional K20 protein may not have been detected using this approach.

Overall our characterisation of K7 knockout mice indicates that K7 is largely dispensable for the development, differentiation and maintenance of those simple epithelia in which it is normally expressed. However, the absence of K7 does appear to affect the normal homeostasis of the bladder urothelium as shown by the increase in urothelial cell proliferation. The urothelium acts as a highly effective barrier by preventing the leakage of urine into the underlying bladder mucosa and is physiologically important in terms of preventing urinary tract infections as well as being clinically important in terms of its susceptibility to carcinoma. Further functional studies using K7 knockout mice may be useful towards understanding the role of K7 within this specialised epithelium in greater detail.

## Supporting Information

Figure S1 **Immunohistochemistry of wildtype (A) and homozygous K7 knockout (B) bladder sections stained with antibodies to the urothelial cell differentiation marker uroplakin 3a.** Notice the intense staining of the intermediate and superficial urothelial cells layers in both samples. m indicates the bladder muscularis; * indicates the lumen of the bladder. Scale bars = 50 µm.
(TIF)

Figure S2 **K18 and K20 expression in the bladder of K7 knockout mice.** Double label immunofluorescence microscopy of wildtype (A-C) and homozygous K7 knockout (D-F) bladder cryosections stained with antibodies to K7 (A, D) and K18 (B, E). Merged images (C, F) show both proteins co-localised at the apical cell membrane of superficial urothelial cells in wildtype mice (arrowheads, C). In homozygous K7 knockout mice, K18 expression appears to be reduced (E) but remains restricted to the superficial cell layer in the absence of K7 (E and F). Wildtype (G-I) and homozygous K7 knockout mice (J-L) bladder cryosections double-labelled with antibodies to K7 (G, J) and K20 (H, K). Merged images are shown in I and L. In the bladder of wildtype mice, K20 is also restricted to the superficial urothelial cells (H) and merged images of G and H shows colocalisation with K7 at the apical cell membrane (arrowheads, I). In homozygous K7 knockout mice, K20 expression (K) appeared similar to wildtype mice (merged image L). Cryosections were counterstained with DAPI. * indicates the lumen of the bladder and m denotes the position of the underlying bladder mucosa. Scale bars = 50 µm.
(TIF)

Figure S3 **Western blots of simple keratin expression in the colon and lung of K7 knockout mice.** A. Coomassie Blue stained SDS-PAGE gel and B. western blots of cytoskeletal extracts of the colon and lung of wildtype (+/+), heterozygous (+/−) and homozygous (–) K7 knockout mice probed with antibodies to K8, K18, K19 and K20. K20 expression was not detected in cytoskeletal extracts from the lung (not shown). M denotes molecular weight standards, sizes in kDa are as indicated.
(TIF)

Figure S4 **K18 expression in the kidney of homozygous K7 knockout mice.** Double-label immunofluorescence microscopy of kidney cryosections from wildtype (A, C, E) and homozygous K7 knockout mice (B, D, F) stained with a rabbit polyclonal antibody to K7 (A, B) and mouse monoclonal antibody Ks18.04 to K18 (C, D). Merged images of A and C and B and D and are shown in panels E and F respectively. In wildtype kidney, both K7 and K18 co-localise and show strong membranous staining of ductal epithelial cells (arrowheads, E). In homozygous K7 knockout mice, the intensity of K18 staining is overall weaker (D) than wildtype kidney (C) although some membranous staining can still be detected (arrowhead, F). Cell nuclei are counterstained with DAPI. Scale bar = 50 µm.
(TIF)

Figure S5 **K7 and K19 expression in the liver of K7 knockout mice.** Double-label immunofluorescence microscopy of liver cryosections from wildtype (A, C, E) and homozygous K7 knockout mice (B, D, F) stained with a rabbit polyclonal antibody to K7 (A, B) and rat monoclonal antibody Troma III to K19 (C, D). Merged images of A and C and B and D and are shown in panels E and F respectively. In wildtype mice, K7 and K19 colocalise and specifically stain the bile duct epithelium (E). In the liver of homozygous K7 knockout mice, K19 staining is not altered by the absence of K7 (D, F). Cell nuclei are counterstained with DAPI. Scale bar = 50 µm.
(TIF)

Table S1 **List of K7 KO tissues examined by H&E staining.**
(DOCX)
