# Supplementary figures and images for: Generation and Characterisation of Keratin 7 (K7) Knockout Mice
Source: PLoS One. 2013 May 31;8(5):e64404. doi: 10.1371/journal.pone.0064404 (PMC3669307; doi:10.1371/journal.pone.0064404)

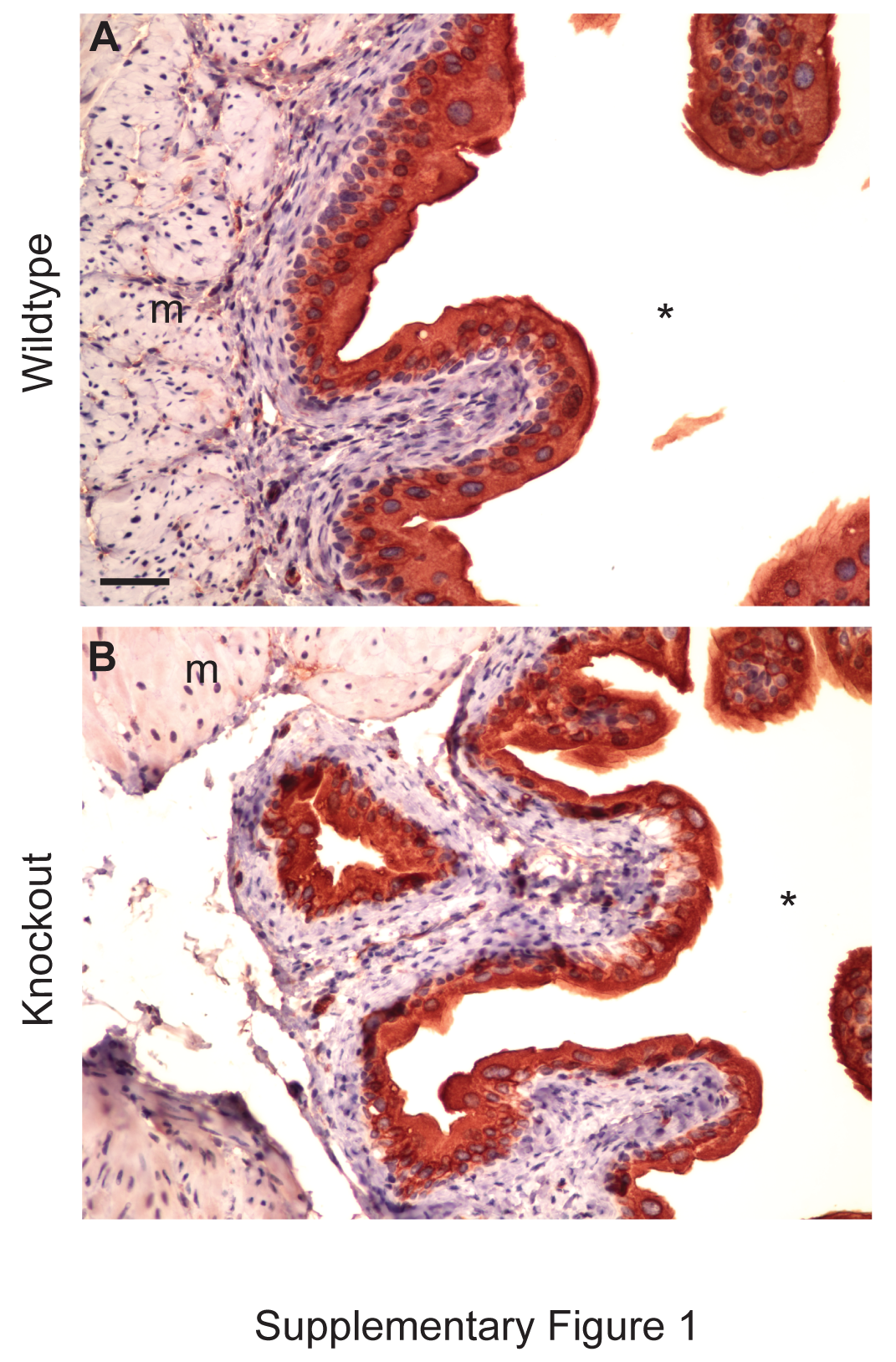

Supplement: Figure S1 — Immunohistochemistry of wildtype (A) and homozygous K7 knockout (B) bladder sections stained with antibodies to the urothelial cell differentiation marker uroplakin 3a. Notice the intense staining of the intermediate and superficial urothelial cells layers in both samples. m indicates the bladder muscularis; * indicates the lumen of the bladder. Scale bars = 50 µm. (TIF) [file pone.0064404.s001.tif]

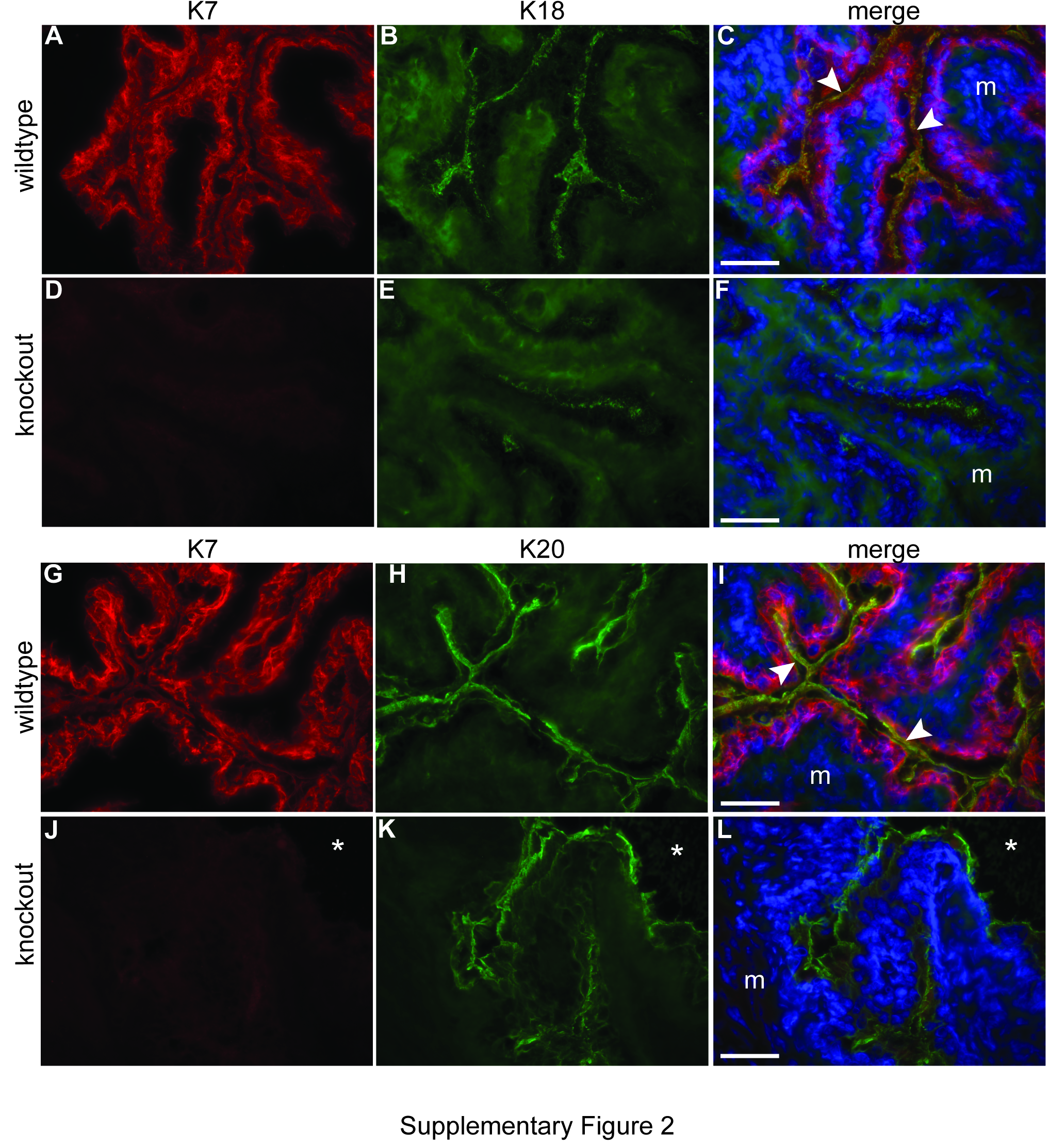

Supplement: Figure S2 — K18 and K20 expression in the bladder of K7 knockout mice. Double label immunofluorescence microscopy of wildtype (A-C) and homozygous K7 knockout (D-F) bladder cryosections stained with antibodies to K7 (A, D) and K18 (B, E). Merged images (C, F) show both proteins co-localised at the apical cell membrane of superficial urothelial cells in wildtype mice (arrowheads, C). In homozygous K7 knockout mice, K18 expression appears to be reduced (E) but remains restricted to the superficial cell layer in the absence of K7 (E and F). Wildtype (G-I) and homozygous K7 knockout mice (J-L) bladder cryosections double-labelled with antibodies to K7 (G, J) and K20 (H, K). Merged images are shown in I and L. In the bladder of wildtype mice, K20 is also restricted to the superficial urothelial cells (H) and merged images of G and H shows colocalisation with K7 at the apical cell membrane (arrowheads, I). In homozygous K7 knockout mice, K20 expression (K) appeared similar to wildtype mice (merged image L). Cryosections were counterstained with DAPI. * indicates the lumen of the bladder and m denotes the position of the underlying bladder mucosa. Scale bars = 50 µm. (TIF) [file pone.0064404.s002.tif]

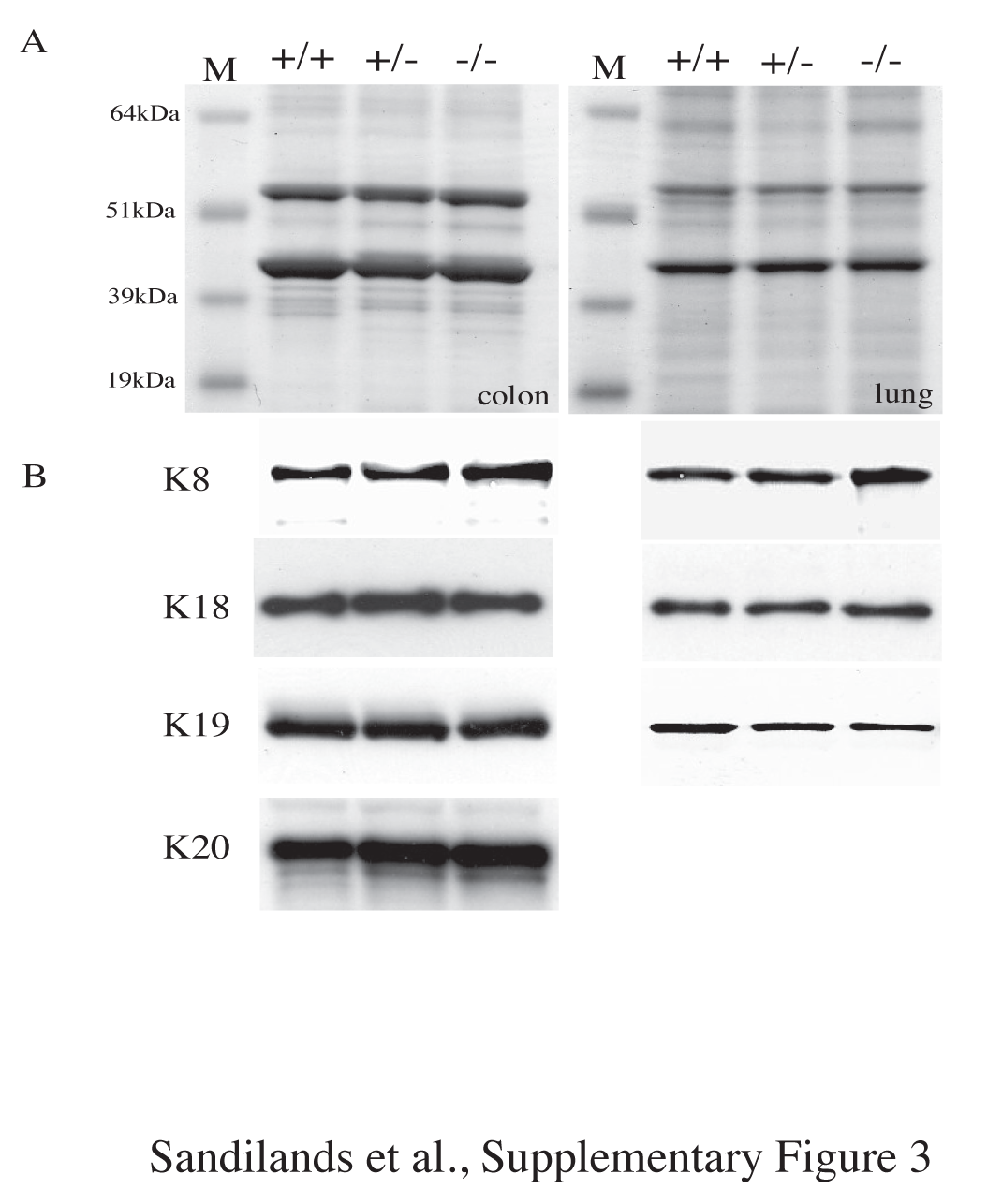

Supplement: Figure S3 — Western blots of simple keratin expression in the colon and lung of K7 knockout mice. A. Coomassie Blue stained SDS-PAGE gel and B. western blots of cytoskeletal extracts of the colon and lung of wildtype (+/+), heterozygous (+/−) and homozygous (–) K7 knockout mice probed with antibodies to K8, K18, K19 and K20. K20 expression was not detected in cytoskeletal extracts from the lung (not shown). M denotes molecular weight standards, sizes in kDa are as indicated. (TIF) [file pone.0064404.s003.tif]

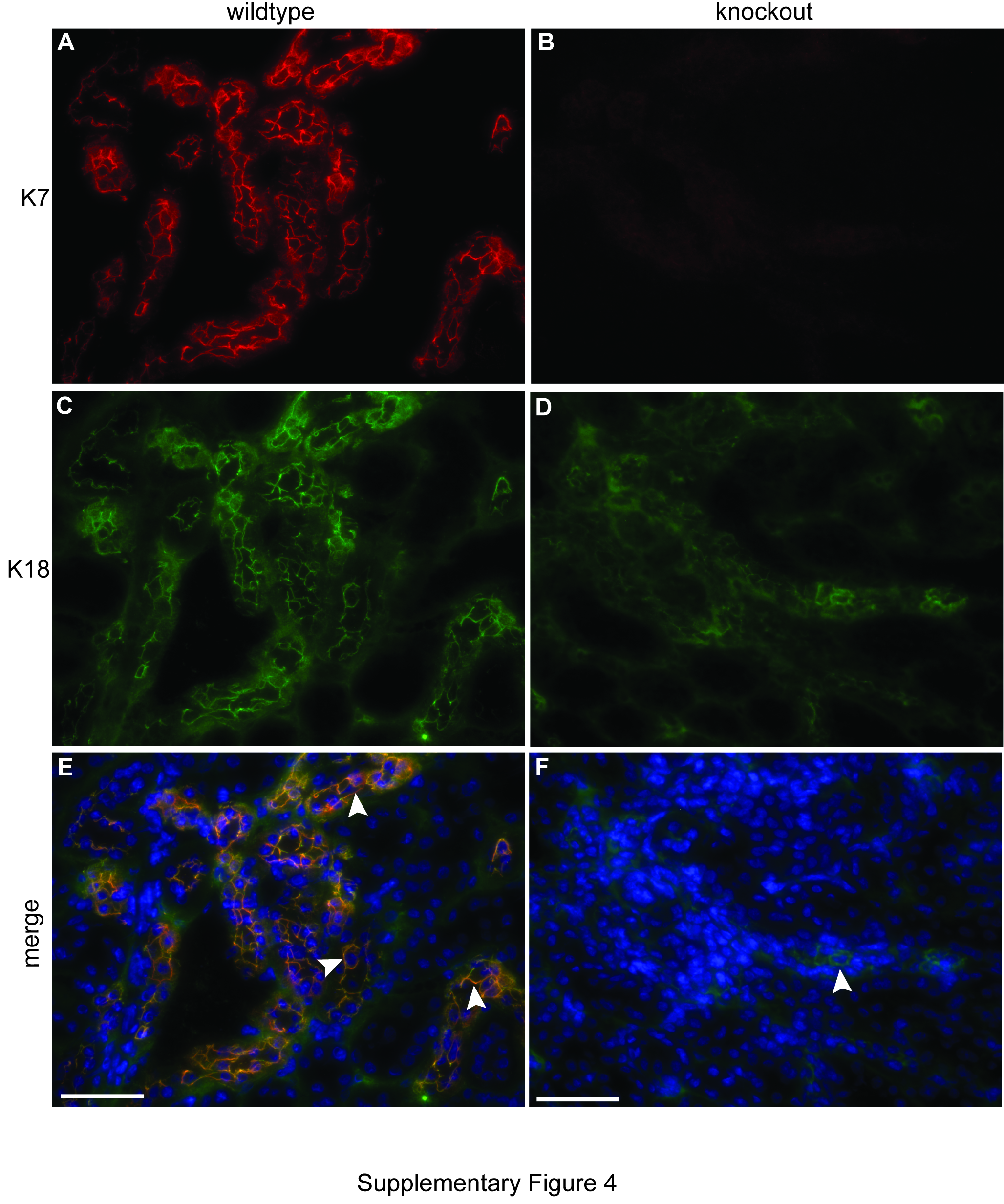

Supplement: Figure S4 — K18 expression in the kidney of homozygous K7 knockout mice. Double-label immunofluorescence microscopy of kidney cryosections from wildtype (A, C, E) and homozygous K7 knockout mice (B, D, F) stained with a rabbit polyclonal antibody to K7 (A, B) and mouse monoclonal antibody Ks18.04 to K18 (C, D). Merged images of A and C and B and D and are shown in panels E and F respectively. In wildtype kidney, both K7 and K18 co-localise and show strong membranous staining of ductal epithelial cells (arrowheads, E). In homozygous K7 knockout mice, the intensity of K18 staining is overall weaker (D) than wildtype kidney (C) although some membranous staining can still be detected (arrowhead, F). Cell nuclei are counterstained with DAPI. Scale bar = 50 µm. (TIF) [file pone.0064404.s004.tif]

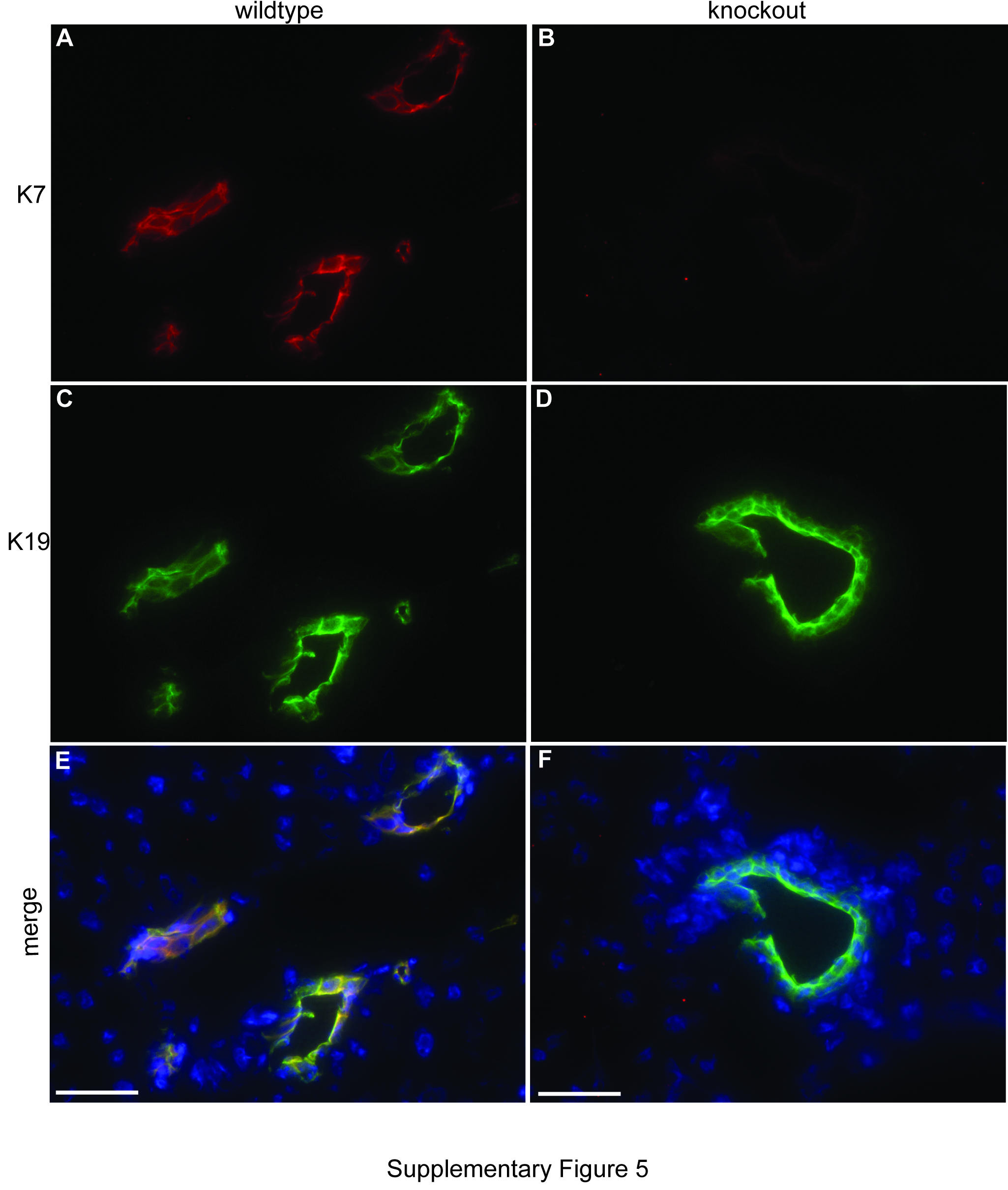

Supplement: Figure S5 — K7 and K19 expression in the liver of K7 knockout mice. Double-label immunofluorescence microscopy of liver cryosections from wildtype (A, C, E) and homozygous K7 knockout mice (B, D, F) stained with a rabbit polyclonal antibody to K7 (A, B) and rat monoclonal antibody Troma III to K19 (C, D). Merged images of A and C and B and D and are shown in panels E and F respectively. In wildtype mice, K7 and K19 colocalise and specifically stain the bile duct epithelium (E). In the liver of homozygous K7 knockout mice, K19 staining is not altered by the absence of K7 (D, F). Cell nuclei are counterstained with DAPI. Scale bar = 50 µm. (TIF) [file pone.0064404.s005.tif]
