# Supplementary material for: Generation and Characterisation of Keratin 7 (K7) Knockout Mice
Source: PLoS One. 2013 May 31;8(5):e64404. doi: 10.1371/journal.pone.0064404 (PMC3669307; doi:10.1371/journal.pone.0064404)
Supplement: Table S1 — List of K7 KO tissues examined by H&E staining. (DOCX) [file pone.0064404.s006.docx]

Sandilands *et al*., Supplementary Table 1.

List of K7 KO tissues examined by H&E staining.

Body system Tissue Site of K7 expression (based on Smith *et al*., 2002.)

*Digestive* Tongue filiform papillae & taste buds

Oesophagus

Stomach transitional cells between stratified & glandular epithelium

Duodenum Brunner’s gland

Colon basal cells in crypts, goblet cells

Liver bile ducts

*Endocrine* Pancreas ductal epithelium

Adrenal gland

*Cardiovascular* Heart not expressed

*Respiratory* Lung alveolar & bronchiolar epithelium

*Genitourinary* Kidney collecting tubules & ducts

Bladder transitional epithelium

Prostate

*Haematopoietic* Spleen not expressed

*Reproductive (male)* Testis not expressed

Seminal vesicle

*Reproductive (female)* Uterus endometrium & endometrial glands

Ovary not expressed

*Integumentary*  Skin (dorsal & ventral) inner root sheath of hair follicle

Nailbed apical cells of nail bed

Paw Merkel cells, sweat glands & ducts

Tail suprabasal cells of parakeratotic scalar region
